# Genome-Wide Duplication of Allotetraploid *Brassica napus* Produces Novel Characteristics and Extensive Ploidy Variation in Self-Pollinated Progeny

**DOI:** 10.1534/g3.120.401493

**Published:** 2020-08-08

**Authors:** Liqin Yin, Zhendong Zhu, Xuan Luo, Liangjun Huang, Yu Li, Annaliese S. Mason, Jin Yang, Xianhong Ge, Yan Long, Jisheng Wang, Qiong Zou, Lanrong Tao, Zeming Kang, Rong Tang, Maolin Wang, Shaohong Fu

**Affiliations:** * College of Life Sciences, Sichuan University, 29 Wangjiang Road, Chengdu, China; † Institute of Crop Research, Chengdu Academy of Agricultural and Forestry Sciences, 200 Nongke Road, Chengdu, China; ‡ Agricultural College, Sichuan Agricultural University, 211 Huimin Road, Chengdu, China; § Plant Breeding Department, Justus Liebig University Giessen, Heinrich-Buff-Ring 26-32, 35396 Giessen, Germany; ** National Key Laboratory of Crop Genetic Improvement, College of Plant Science and Technology, Huazhong Agricultural University, Wuhan, China; †† Biotechnology Research Institute, Chinese Academy of Agricultural Sciences, 12 Zhongguancun South Street, Beijing, China

**Keywords:** allopolyploid, *Brassica napus*, genome instability, mitotic abnormality, pollen aperture, polyploid evolution

## Abstract

Whole genome duplications (WGDs) have played a major role in angiosperm species evolution. Polyploid plants have undergone multiple cycles of ancient WGD events during their evolutionary history. However, little attention has been paid to the additional WGD of the existing allopolyploids. In this study, we explored the influences of additional WGD on the allopolyploid *Brassica napus*. Compared to tetraploid *B. napus*, octoploid *B. napus* (AAAACCCC, 2*n* = 8*x* =76) showed significant differences in phenotype, reproductive ability and the ploidy of self-pollinated progeny. Genome duplication also altered a key reproductive organ feature in *B. napus*, that is, increased the number of pollen apertures. Unlike autopolyploids produced from the diploid *Brassica* species, the octoploid *B. napus* produced from allotetraploid *B. napus* had a relatively stable meiotic process, high pollen viability and moderate fertility under self-pollination conditions, indicating that sub-genomic interactions may be important for the successful establishment of higher-order polyploids. Doubling the genome of *B. napus* provided us with an opportunity to gain insight into the flexibility of the *Brassica* genomes. The genome size of self-pollinated progeny of octoploid *B. napus* varied greatly, and was accompanied by extensive genomic instability, such as aneuploidy, mixed-ploidy and mitotic abnormality. The octoploid *B. napus* could go through any of genome reduction, equilibrium or expansion in the short-term, thus providing a novel karyotype library for the *Brassica* genus. Our results reveal the short-term evolutionary consequences of recurrent polyploidization events, and help to deepen our understanding of polyploid plant evolution.

Polyploidy or whole genome duplication (WGD) has long been considered a prominent force driving evolution of angiosperm species (Casacuberta *et al.* 2016; Leitch and Leitch 2008; Soltis *et al.* 2009; Jiao *et al.* 2011). Advances in sequencing have revealed a series of ancient WGDs throughout the evolutionary history of angiosperms (Jiao 2018; Wendel *et al.* 2016; Jiao *et al.* 2011; Ren *et al.* 2018). All extant seed plants have experienced at least one cycle of WGD in their evolutionary history (Jiao *et al.* 2011). More than a third of present angiosperm plants are polyploids (Wood *et al.* 2009), and approximately 13% of diploid plant species populations may harbor unnamed polyploid cytotypes (Barker *et al.* 2016). Accumulating evidence suggests that polyploidy has played a significant role in phenotypic innovation, species diversification, facilitation of biodiversity, and adaptation to broader ecological environments (Soltis *et al.* 2009; Schranz *et al.* 2012; Ramsey and Schemske 1998; Hohmann *et al.* 2015; Van de Peer *et al.* 2017; Mandáková *et al.* 2017; Jiao 2018; Soltis and Soltis 2016; Edger *et al.* 2015; Tank *et al.* 2015). Polyploidy may also facilitate species survival and radiation during periods of rapid ecological change (Vanneste *et al.* 2014; Jiao 2018; Cai *et al.* 2019; Chao *et al.* 2013).

*Brassicaceae* is one of the most abundant angiosperm families and includes model plant *Arabidopsis thaliana* and economically important *Brassica* oil-crops and vegetables (Wang *et al.* 2011). There is strong evidence that WGDs frequently occur in the evolutionary history of the *Brassicaceae* family (Soltis *et al.* 2009; Van de Peer *et al.* 2009; Schranz *et al.* 2012; Jiao *et al.* 2011; Romanpalacios *et al.* 2019; Mandáková *et al.* 2017; Ren *et al.* 2018). All *Brassicaceae* species share a common ancient WGD event occurring around the origin of the family (Schranz and Mitchell-Olds 2006; Schranz *et al.* 2012). *Arabidopsis thaliana* has undergone at least five rounds of WGD across its evolutionary history (Jiao *et al.* 2011). All *Brassica* species share the complex evolutionary history of *Arabidopsis thaliana* but with the addition of a lineage-specific whole-genome triplication (Couvreur *et al.* 2010; Lysak *et al.* 2005; Wang *et al.* 2011; Yang *et al.* 2016). Diploid species *Brassica rapa* (AA, 2*n* = 20) and *Brassica oleracea* (CC, 2*n* = 18) are considered mesohexaploids (Parkin *et al.* 2014; Liu *et al.* 2014; Wang *et al.* 2011). *Brassica napus* (AACC, 2*n* = 4*x* = 38) is a recent allopolyploid species formed about 7500 years ago by hybridization between *B. rapa* and *B. oleracea* (Chalhoub *et al.* 2014). *B. napus* has undergone a predicted 72 × polyploidization since the origin of the angiosperms (Chalhoub *et al.* 2014). It is widely accepted that recurrent polyploidization has driven evolution of key traits, species diversity, and profound evolutionary novelty in the *Brassica* lineage (Schranz *et al.* 2012; Romanpalacios *et al.* 2019; Van de Peer *et al.* 2009; Hofberger *et al.* 2013; Edger *et al.* 2015; Wang *et al.* 2011).

*B. napus* is a classic example of the importance of WGD in plant evolution, and is considered an ideal system for investigation of the consequences and mechanisms of *de novo* and recurrent polyploidization (Jenczewski and Alix 2004; Chalhoub *et al.* 2014; Szadkowski *et al.* 2010; Ferreira de Carvalho *et al.* 2019; Mason and Snowdon 2016). Recent analysis suggests that recurrent WGDs may cyclically provide novel genetic resources for evolution and diversification, because duplicated sequences from ancient WGD tend to be lost in species that experienced additional recent WGD (Jiao 2018; Ren *et al.* 2018). However, although polyploidization remains a highly dynamic, active and ongoing process in nature (Leitch and Leitch 2008; Comai 2005; Alix *et al.* 2017), it is largely unclear whether recent allopolyploid species can benefit from an additional round of WGD, or if novel traits will be derived in such a scenario. Recently, we tried to double the genome of allotetraploid *B. napus*, and found that the octoploid *B. napus* has a novel function (Fu *et al.* 2018): when used as a pollen donor, octoploid *B. napus* induces tetraploid *B. napus* (female) to produce maternal doubled-haploid progeny with almost all genetic material coming from the female parent (Fu *et al.* 2018; Fu *et al.* 2019). This novel method allows production of homozygous *B. napus* lines within two years, without the necessity of using microspore culture (Fu *et al.* 2018; Fu *et al.* 2019). Nevertheless, the influences of WGD on the physiological and reproductive characteristics of *B. napus* are as yet largely unknown and worth exploring. Against the background of global climate change and the accelerated rate of plant species extinction (Humphreys *et al.* 2019; Knapp *et al.* 2020), doubling the genome of allopolyploid rapeseed may innovate germplasm resources for further genomic and genetic research. Here we have investigated the vegetative, reproductive, and karyotypic effects of duplicating an allotetraploid genome, and assessed the vigor, fitness and fertility of octoploid *B. napus*, as well as the short-term evolutionary trend of higher-order newly-formed polyploids, offering a unique perspective into the constraints and mechanisms operating in the evolution of the angiosperms.

## Materials And Methods

### Plant materials

Two octoploid rapeseed lines (Y3380 and Y3560, AAAACCCC, 2*n* = 8*x* = 76) were artificially synthesized by doubling the genome of allotetraploid *B. napus* (Figure S1). The octoploid *B. napus* used here was the seventh self-pollinated generation of the newly resynthesized octoploid rapeseed. P3-2 (AACC, 2n = 4*x* = 38) was a common parent of the two octoploid *B. napus* lines. The conventional *B. napus* variety ZS11 (2*n* = 4*x* = 38) which has been widely cultivated in China was used as a control. All plant materials were grown under the same conditions at the experimental field in Wenjiang (E103.83, N30.70), Chengdu, China. The plants were self-pollinated by bagging in the field to obtain self-pollinated seeds.

### Fluorescent in situ hybridization

Fluorescence *in situ* hybridization (FISH) analysis was carried out according to protocols detailed in (Cui *et al.* 2012). The probe used for *in situ* hybridization was BAC BoB014O06, which contains C-genome specific repeat elements (Xiong *et al.* 2011) and was labeled with biotin-11-dCTP. The A-genome was visualized with DAPI (4’,6-diamidino-2-phenylindole) as a background stain under UV fluorescence. Photographs were taken with a fluorescence microscope (ZEISS AX10) and image processing was performed using Adobe Photoshop CS6.

### Phenotypic observations

Phenotypic characteristics of the rapeseed materials were observed and photographed by SLR camera (E0S 200D, Cannon). The plant height, length of main inflorescence, primary branch number, number of effective pods per plants, pod length, number of seeds in a pod, number of ovules in a pod, 1000-seed weight and the germination rate of self-pollinated seeds of 30 octoploid *B. napus* plants were counted and recorded.

### Pollen viability estimation

Mature pollen viability was estimated using the Alexander staining method (Peterson *et al.* 2010), and pollen were photographed using a light microscope.

### Scanning electron microscopy observation

The morphology of the pollen grains was observed by a cold field emission scanning electron microscope (SU8010, Hitachi). Detailed steps were as follows: mature anthers were fixed with 4% glutaraldehyde (pH 6.8) for 12 hr and then rinsed 3 times with 0.1M phosphate buffer (pH 7.4) for 15 min each time; samples were then fixed with 1% citric acid·0.1 M phosphate buffer (pH 7.4) at 20° for 1-2 h, and then rinsed 3 times with 0.1 M phosphate buffer (pH 7.4) for 15 min each time. The pollen was placed in 30%, 50%, 70%, 80%, 90%, 95%, 100%, and 100% ethanol in a stepwise dehydration for 15 min each, followed by incubation in isoamyl acetate for 15 min. The treated anthers were placed on a CO_2_ critical point dryer for drying, and the sample was adhered to the conductive carbon film with double-sided tape, then placed on the ion sputter sample stage for about 30 sec and photographed.

### In vitro pollen tube germination

The *in vitro* germination experiment of mature pollen grains was carried out according to the procedures detailed in Feng (2017).

### Meiotic behavior observation

Observations of meiotic behavior were carried out according to the procedures detailed in (Li *et al.* 1995). Young buds (2-3 mm long) were collected in Carnoy’s fixative (ethanol: glacial acetic acid, 3:1, v/v) in the morning, and fixed for 24 h at room temperature. Young anthers were dissected out, incubated in 1 M hydrochloric acid solution at 60° for 6-8 min, removed and placed on a glass slide, squeezed gently to release pollen mother cells and sporads into Carbol fuchsin solution, and then observed under an optical microscope.

### Somatic chromosome counting

Young ovaries were used to count the somatic chromosome number (Zhou *et al.* 2016). The ovaries were treated with 0.002 mol/L 8-hydroxyquinoline solution for 3 hr in the dark, then transferred to Carnoy’s fixative (ethanol: glacial acetic acid, 3:1, v/v) for more than 24 hr. Cytogenetic observation was carried out according to the procedures detailed in Li *et al.* (1995).

### Ploidy determination by flow cytometry

Flow cytometry analysis was performed to assess the plant DNA content. A total of 414 individuals were randomly selected from the self-pollinated offspring of 19 octoploid Y3380 plants, and 588 individuals derived from 30 octoploid Y3560 plants were randomly selected. Details of procedures were as referred to in (Dolezel *et al.* 2007), with slight modifications. Fresh young leaves were taken between 9 am to 11 a.m, washed with distilled water and dried with filter paper. Leaves with a diameter of 0.5 cm was taken using a hole punch and placed in a pre-cooled Petri dish. Subsequently, 0.5 ml of pre-cooled LB01 cell lysis buffer (15 mM Tris, 2 mM disodium edetate, 0.5 mM spermine tetraamine, 80 mM potassium chloride, 20 mM sodium chloride, 0.1% (v/v) polyethylene glycol octylphenyl ether and 15 mM β-mercaptoethanol, pH 7.5, 0.22 μm filtered) was added. The leaves were chopped quickly into pieces with a sharp blade, filtered with a 75 μm filter, and then stained with 1 mL PI solution (5% propidium iodide and 5% RNA enzyme) for 30 min in the dark, then measured by flow cytometry (Accuri C6 Plus, BD). At least 20000 cells were collected from one sample. Data were analyzed using AccuriC software. The formula for calculating the approximate chromosome number of the samples was as follows (Dolezel *et al.* 2007).

$$S\text{ample chromosome No}.=\text{reference chromosome No}.\times \frac{mean\;position\;of\;the\;\text{G1 sample peak}}{mean\;position\;of\;the\;\text{G1 reference peak}}$$

### Data availability

File S1 contains two sets of flow cytometry histograms of octoploid *B. napus* (Y3380 plants and Y3560 plants). File S2 contains two sets of flow cytometry histograms of tetraploid *B. napus* (ZS11 and P3-2). File S3 contains two sets of flow cytometry histograms of the self-pollinated individuals derived from octoploid *B. napus*. All flow cytometry fluorescence histograms were output directly from the AccuriC software. Supplemental Figures contains Figures S1-S12. Table S1 contains the fluorescence intensity values of the mitotic G1 phase of octoploid and tetraploid *B. napus*. Table S2 contains phenotypic data for octoploid and tetraploid *B. napus*. Table S3 contains the fluorescence intensity values of the mitotic G1 phase of self-pollinated individuals derived from octoploid *B. napus*. Supplemental material available at figshare: https://doi.org/10.25387/g3.12593627.

## Results

### Chromosomal constitution of octoploid rapeseed

**Figure 1:**
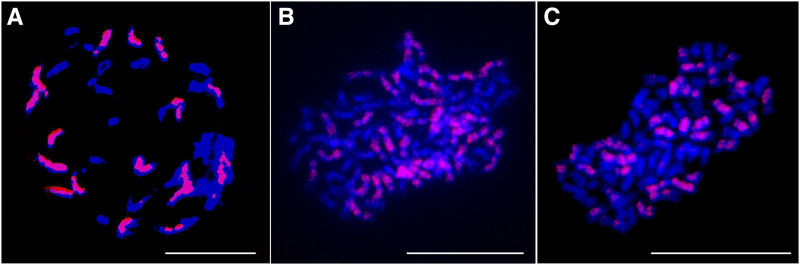
FISH analysis of mitotic cell in rapeseed materials with different ploidy levels. Blue signals were from DAPI staining and red signals were from a C-genome-specific probe (BAC BoB014O06). (A) ZS11, tetraploid, 2*n* = 38 (20A + 18C). (B) Y3380, octoploid, 2*n* = 76 (40A+36C). (C) Y3560, octoploid, 2*n* = 76 (40A + 36C). Bar: 10 μm.

The octoploid rapeseed obtained by doubling the genome of tetraploid *B. napus* had 76 chromosomes in somatic cells (Fu *et al.* 2018). FISH results further confirmed that the somatic cells of the two octoploid rapeseeds contained ∼36 C_n_ chromomes and ∼40 A_n_ chromosomes (Figure 1), with a chromosomal composition of AAAACCCC, confirming that the octoploid rapeseed is actually octoploid *B. napus*. Flow cytometry analysis showed that the fluorescence intensity value of the mitotic G1 phase of octoploid *B. napus* was around 865,000, and that of tetraploid *B. napus* was about 423,000 (Table S1, File S1-S2).

### WGD-driven changes in phenotypes and physiology

**Figure 2:**
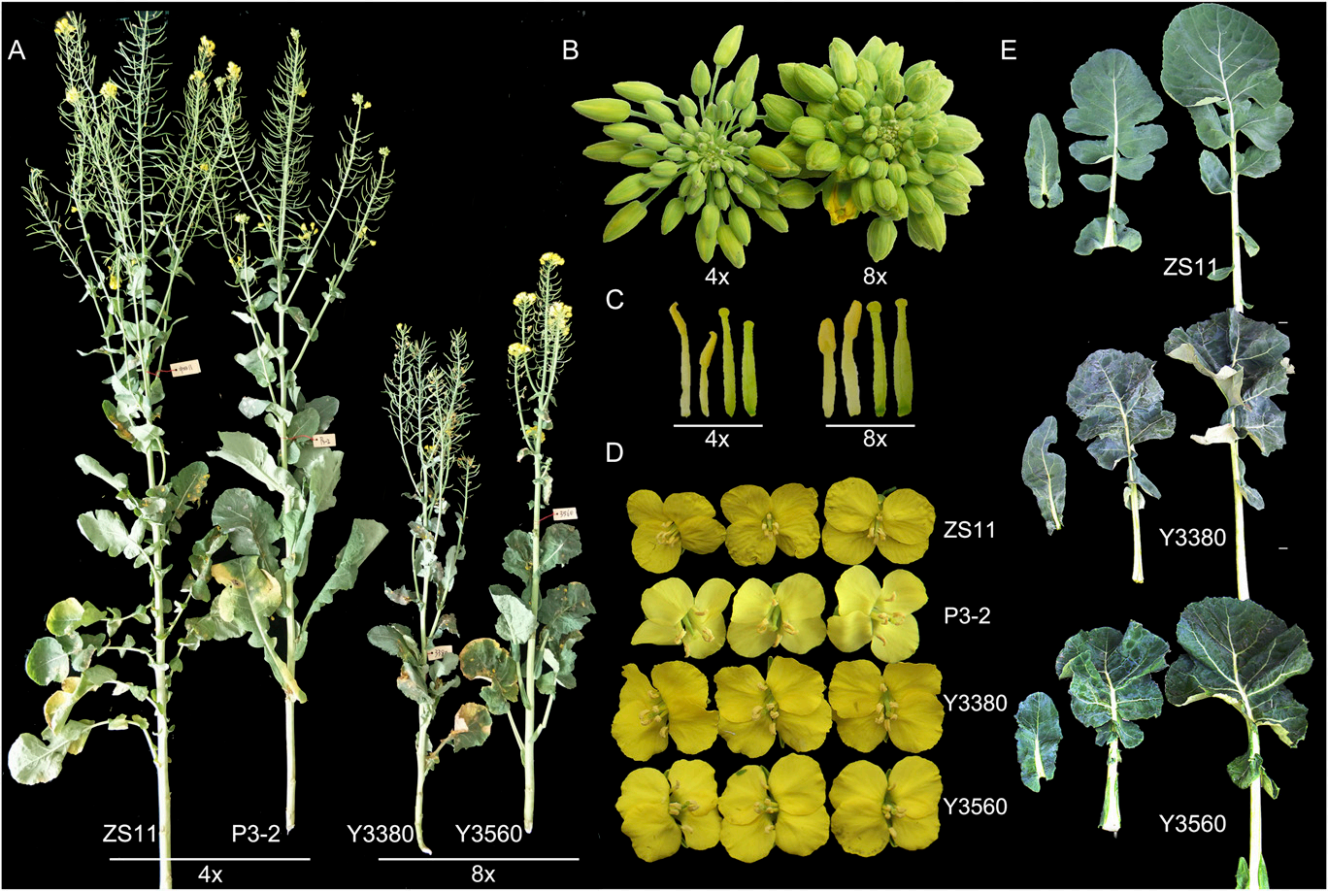
Morphological characteristics of tetraploid *B. napus* (ZS11 and P3-2) and octoploid *B. napus* (Y3380 and Y3560) plants. (A) Plant morphology at the silique formation stage. (B) Flower buds. (C) Stamens and flower styles. (D) Fully-opened flowers. (E) Leaves.

**Figure 3:**
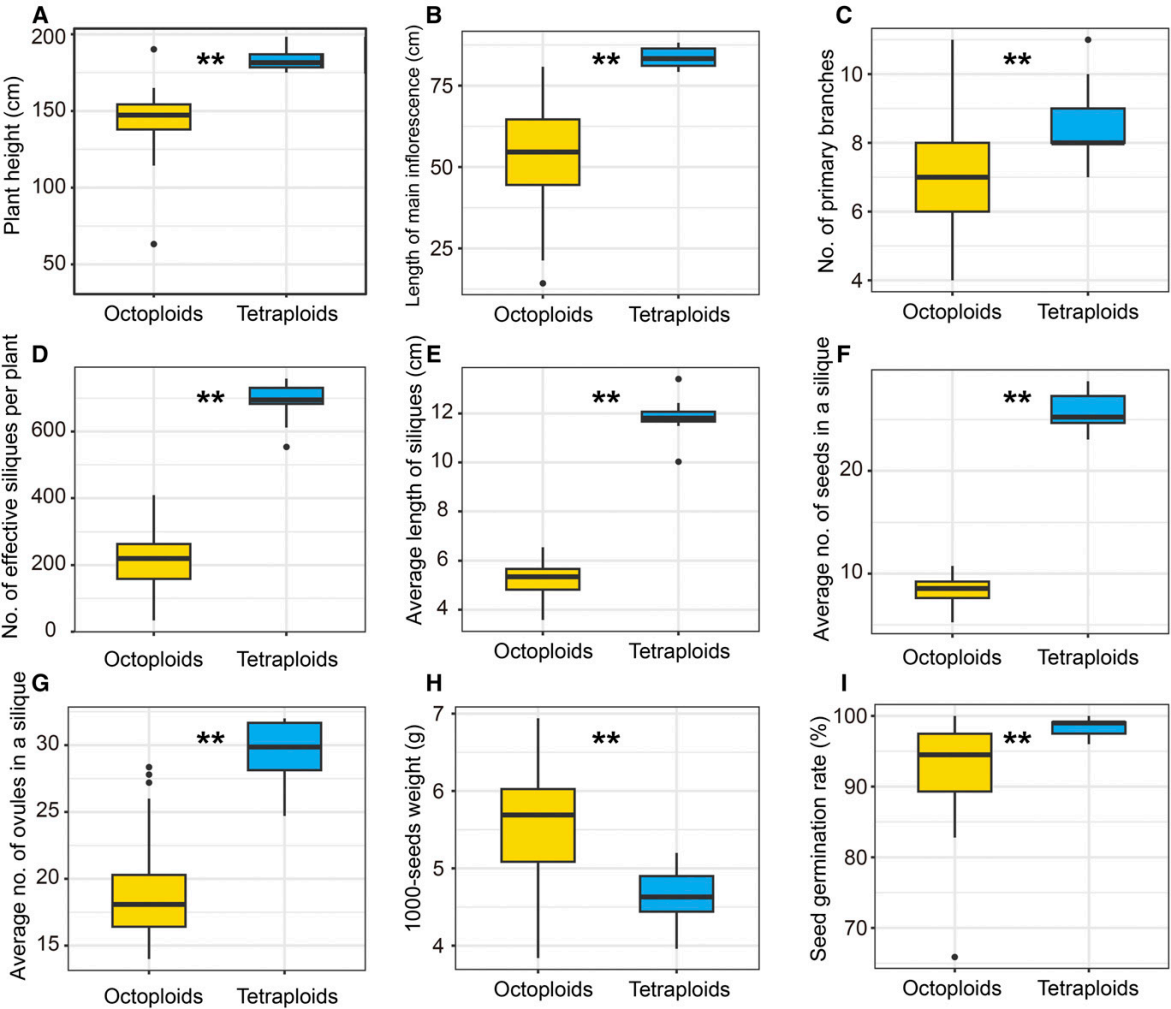
Boxplots of agronomic traits of octoploid and tetraploid *B. napus*. The octoploid plants were from the Y3560 population and the tetraploid control was ZS11. (A) Plant height. (B) Length of the main inflorescence of the rapeseed plant. (C) Number of primary branches per plant. (D) Number of effective siliques (pods containing seeds) per plant. (E) Average length of self-pollinated siliques (10 siliques per plant). (F) Average seed number per silique (10 siliques per plant). (G) Average ovule number per siliques (10 siliques per plant). (H) 1000-seed weight for self-pollinated seeds. (I) Seed germination rate of the self-pollinated seeds. ** indicated significant difference (*P* < 0.01, by Wilcoxon’s rank sum test) between groups.

Although the appearance of octoploid *B. napus* was roughly similar to that of tetraploid *B. napus* (Figure 2), WGD caused phenotypic and physiological differences between the two *B. napus* types. The octoploid *B. napus* was shorter (Figure 2A), with plant height ranging from 62 to 189 cm (average 146 cm), while the average plant height of tetraploid *B. napus* was 183 cm (Figure 3A, Table S2). The average length of the main inflorescence of octoploid *B. napus* decreased by 36% relative to the tetraploid (Figure 3B, Table S2), and the average number of primary branches of octoploid *B. napus* showed a reduction of about 20% compared to that of the tetraploid (Figure 3C, Table S2). Additionally, compared with tetraploid *B. napus*, the octoploid *B. napus* grew slower at the seedling stage (Figure S2), the leaves were thicker and deformed (Figure S3), and the floral organs were slightly larger (Figure 2B-D). Novel phenotypic variation was also prevalent in the octoploid *B. napus* population (Figure S3-S4).

### Ploidy-induced variation in male reproductive organs

**Figure 4:**
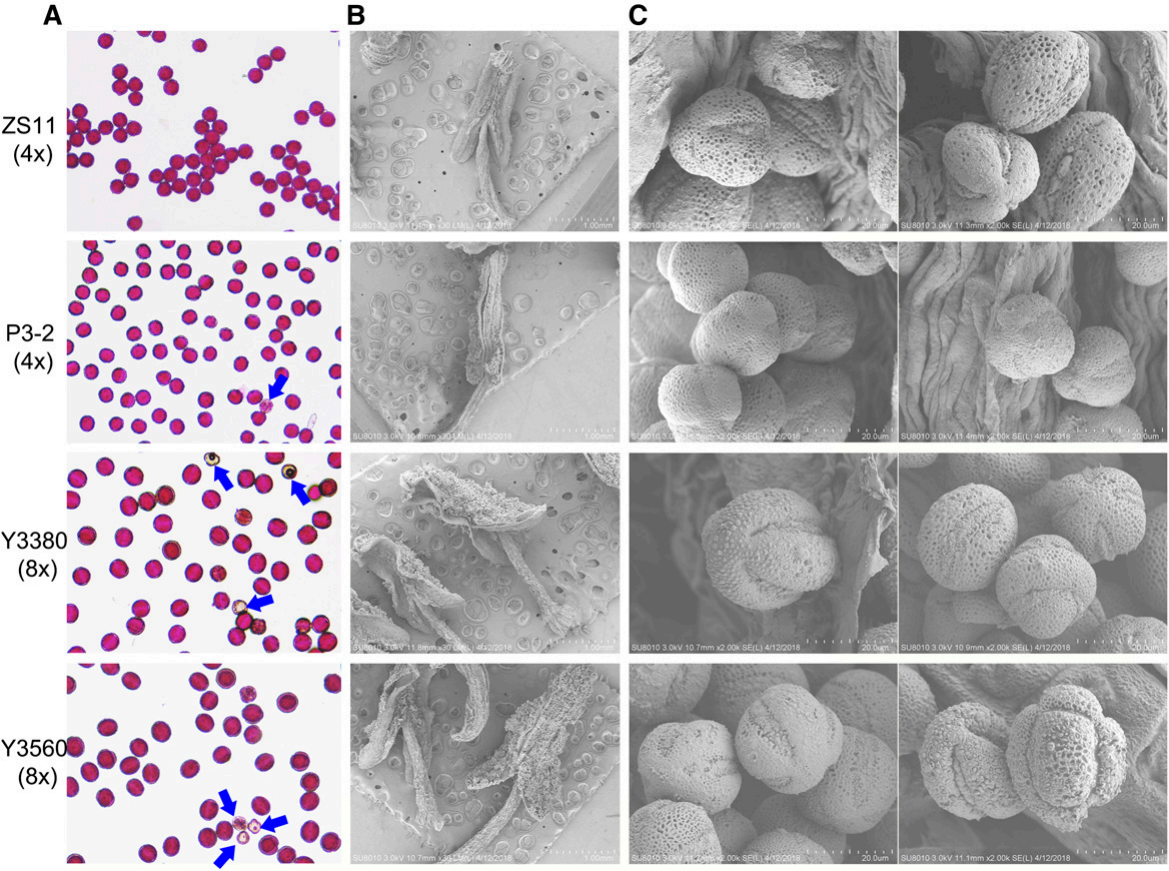
Viability detection and morphological observation of pollen grains. In the horizontal direction, the samples were ZS11, P3-2, Y3380 and Y3560 in order from top to bottom. Pollen viability was detected by Alexander staining method (A). Non-viable pollen grains were indicated with blue arrows. Morphology of anther (B) and pollen grains (C) were observed by scanning electron microscope.

Pollen viability of the octoploid *B. napus* was generally extremely high, up to 90%, only slightly lower than that of the tetraploids (Figure 4A). Compared to tetraploid *B. napus*, octoploid *B. napus* had larger anthers, and each anther contained more pollen grains (Figure 4B). Scanning electron microscopy observation of pollen grains revealed that more than 98% of the octoploid *B. napus* pollen grains had mutated exine patterns, with an increase in the number of pollen apertures to four (Figure 4C) (natural *Brassica* plants have only three pollen apertures per pollen grain). Some octoploid *B. napus* pollen had apertures asymmetrically distributed on the pollen wall (Figure 4C). *In vitro* pollen germination assays showed that the pollen grains of octoploid *B. napus* might grow faster than those of tetraploid *B. napus* (Figure S5).

### Meiotic process of octoploid B. napus

**Figure 5:**
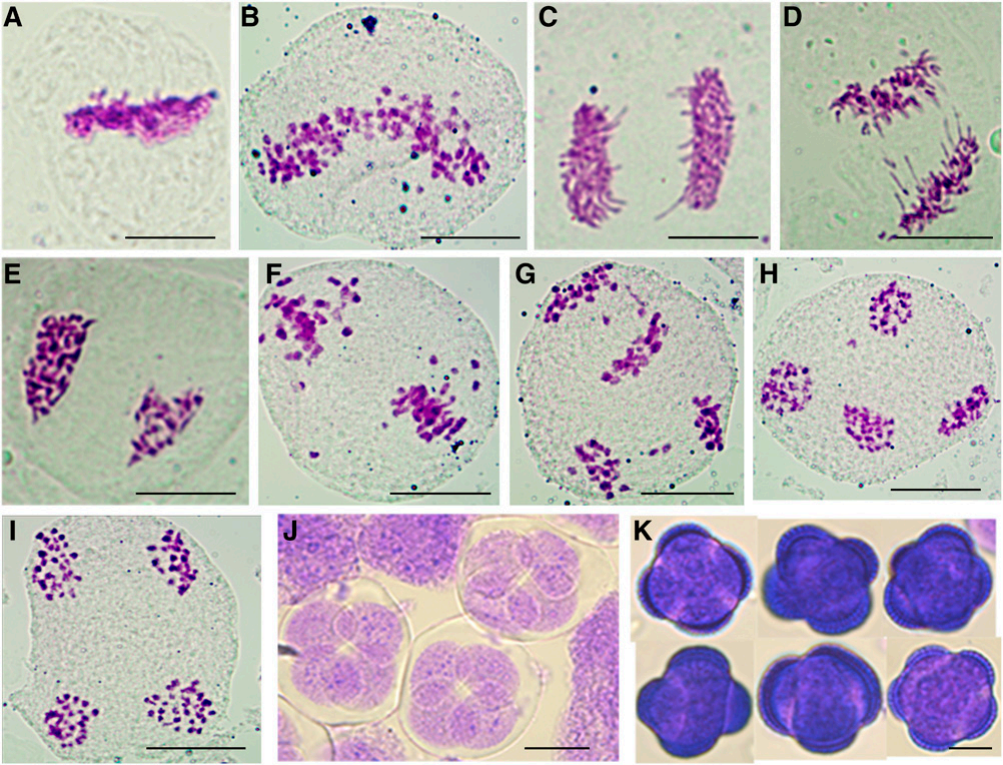
Observation of meiosis process in pollen mother cells of octoploid *Brassica*. (A-B) Metaphase I. (C-D) Anaphase I. (E-F) Telophase I. (G) Anaphase II. (H-I) Telophase II. (J) Tetrads. (K) Mature pollen grains. Bar: 10 μm.

In order to explore the mechanisms underlying high pollen viability in octoploid *B. napus*, the meiotic process of the pollen mother cells was studied. Many abnormal chromosomal behaviors were detected, including multivalent chromosome pairing, irregular chromosome arrangements during metaphase, lagging chromosomes, chromosome bridges and chromosome breakages at anaphase, and unequal chromosome distribution at telophase (Figure 5). However, some cells still had relatively normal meiotic processes (Figure 5I). It is worth noting that after telophase II, more than 99% of pollen mother cells were divided into four parts, eventually forming normal tetrads. The tetrads finally formed regular pollen grains with four apertures, and a few pollen grains had five or more apertures (Figure 5K). Overall, there were certain abnormalities during meiosis in octoploid *B. napus*, but the general process was relatively normal, which may ultimately promote the formation of viable pollen grains.

### Fertility of octoploid B. napus

**Figure 6:**
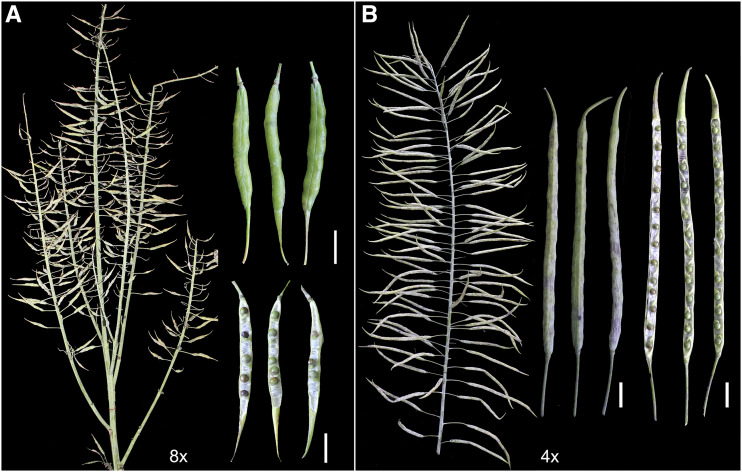
Self-pollinated siliques of octoploid (A) and tetraploid (B) *B. napus* at the fruiting stage. Seed setting, siliques, and seeds in a dissected silique were shown. Bar: 1 cm.

The number of self-pollinated siliques harvested from octoploid *B. napus* ranged from 34 to 409, with an average of 216, while tetraploid *B. napus* produced an average of 692 siliques per plant (Figure 3D). The relatively small number of effective siliques (with normal seeds inside) on an octoploid *B. napus* plant was partly due to the fact that some flowers could not develop into effective siliques (Figure 6A). The siliques derived from self-pollination of octoploid *B. napus* were significantly (*P* < 0.05) shorter than those of tetraploid *B. napus*: the average length of the former was about half that of the latter (Figure 3E, Figure 6). Additionally, the average number of seeds in a silique derived from self-pollination of octoploid *B. napus* was between 5.3 and 10.8, with a median of 8.6, while that of tetraploid *B. napus* was about 25.9 (Figure 3F, Figure 6). In addition to the small number of ovules in a pistil (Figure 3G) compared with tetraploid *B. napus*, the reasons for the reduced fertility of octoploid *B. napus* included the following factors: some pollen grains could not germinate on the stigma (Figure S6); pollen tubes could not elongate properly (Figure S6); the number of pollen tubes entering the style was reduced (Figure S6); some pollen tubes could not accurately identify the ovule and release sperm (Figure S6); and some embryos aborted after fertilization during the post-zygotic development phase (Figure S7). Although the seeds from self-pollination of octoploid *B. napus* varied in size and shape (Figure S8), and their average thousand-seed weight ranged from 3.8 to 6.9 g (Figure 3H, Table S2), they had a high seed germination rate (average 93%) (Figure 3I). Overall, the octoploid *B. napus* had an intermediate level of reproductive capability.

### DNA content of self-pollinated progeny of octoploid B. napus

**Figure 7:**
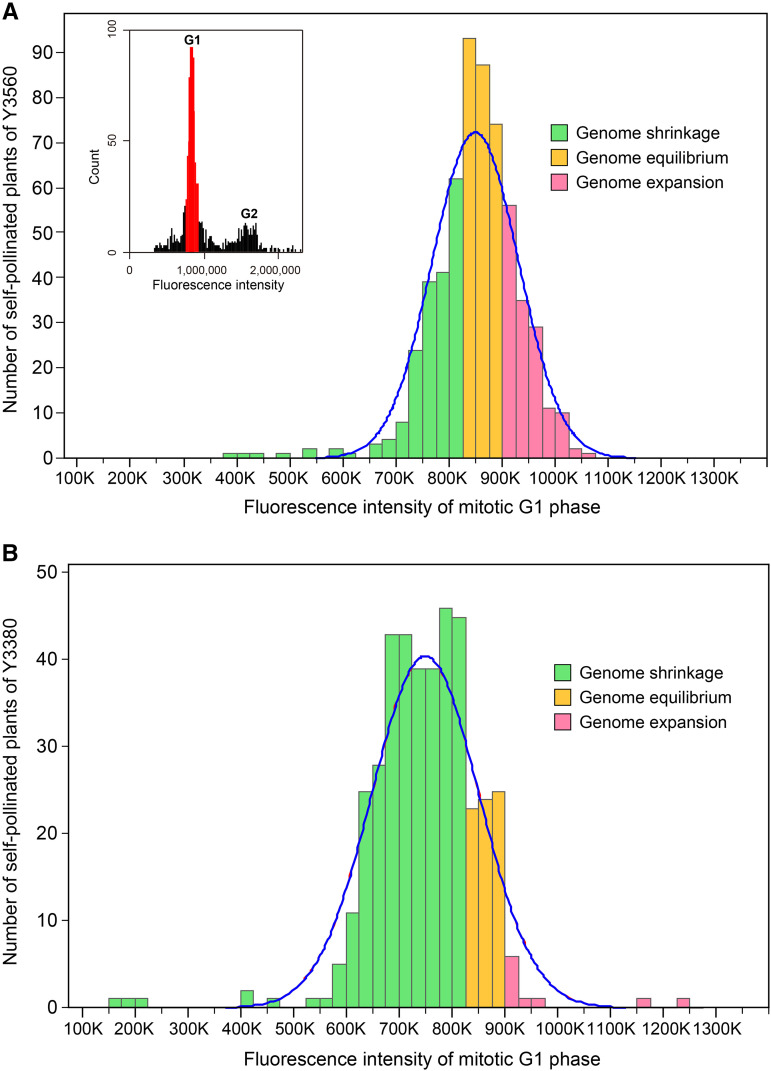
Distribution of DNA content in self-pollinated plants derived from octoploid *B. napus*. A total of 588 self-pollinated individuals of 30 octoploid Y3560 plants were tested (A), and 414 inbred individuals of 19 octoploid Y3380 plants were detected (B). DNA content was detected by flow cytometry, and expressed as fluorescence intensity of the mitotic G1 phase. The green columns represent plants with DNA content lower than that of their octoploid parent, that is, the genome size of these progeny has shrunk. The orange columns represent individuals with DNA content close to that of the octoploid *B. napus* at a 99% confidence level (genome equilibrium). The pink columns represent plants with expanded genome size compared to the octoploid parent. The blue solid line indicates the normal distribution curve.

Flow cytometry analysis showed that the DNA content of the self-pollinated progeny of octoploid *B. napus* varied greatly and was normally distributed (Figure 7). Of the 558 self-pollinated offspring of octoploid Y3560, about 237 individuals had DNA content close to that of octoploid *B. napus* (with a confidence level of 99%), 210 individuals had lower DNA content than the octoploid parent, and the genome size of the remaining 141 individuals was larger than that of the octoploids (Figure 7A, Table S3). Among the 414 self-pollinated progeny of octoploid Y3380, approximately 70 individuals had DNA content close to that of octoploid *B. napus* (with a confidence level of 99%), while 335 individuals had DNA content lower than that of the octoploid parent, and only 9 individuals had a genome size larger than that of the octoploid parent (Figure 7B, Table S3). In the Y3560 offspring population, the maximum DNA content was 2.7 times the minimum DNA content; and in the Y3380 offspring population, this maximum DNA content was 7.3 times the minimum DNA content (Table S3). Due to the linear relationship between the DNA content and the number of somatic chromosomes (Figure S9), we speculated that the somatic chromosome number of the self-pollinated offspring derived from octoploid *B. napus* could be up to 100 (Table S3). Additionally, FISH analysis showed that the C sub-genome chromosomes from octoploid *B. napus* might be more vulnerable to loss in the self-pollinated progeny than the A sub-genome chromosomes (Figure S10). The self-pollinated progeny generation of octoploid *B. napus* had an extensive range of ploidy variation, where ploidy was not only maintained around the octoploid level in some individuals, but also reduced or expanded in others.

### Abnormal mitosis in the self-pollinated progeny of octoploid B. napus

**Figure 8:**
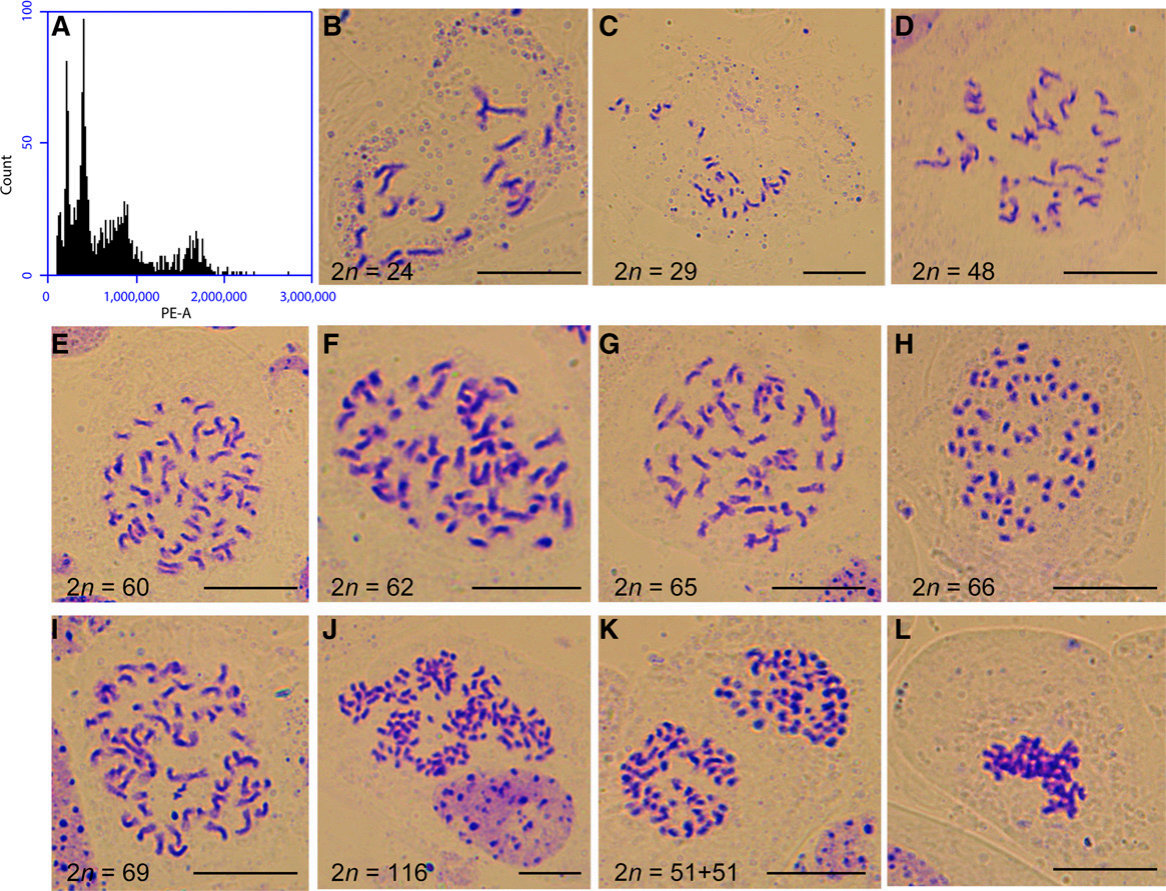
Flow cytometry analysis and somatic chromosome observation of a mixed-ploidy plant derived from octoploid Y3380. (A) Flow cytometry histogram. (B) One cell with 2*n* = 24. (C) One cell with 2*n* = 29. (D) One cell with 2*n* = 48. (E) One cell with 2*n* = 60. (F) One cell with 2*n* = 62. (G) One cell with 2*n* = 65. (H) One cell with 2*n* = 66. (I) One cell with 2*n* = 69. (J) One cell with 2*n* = 116. (K) One cell with 2*n* = 102, telophase. (L) Abnormal chromosomal arrangement. Bar: 10 μm.

**Figure 9:**
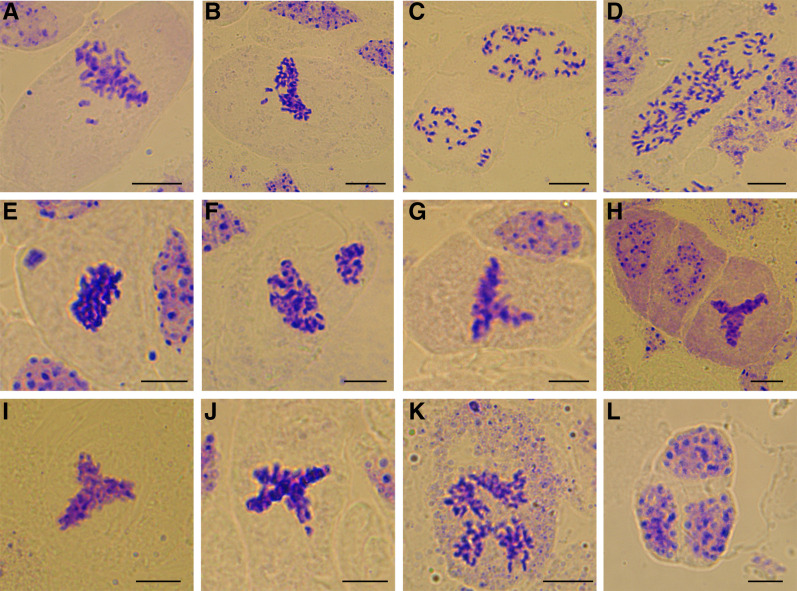
Mitotic abnormalities of mixed-ploidy plants. (A-B) Lagging chromosomes during mitotic metaphase. (C) Chromosomes were unevenly distributed into daughter cells in late mitosis. (D) One cell with too many chromosomes, 2n ≈ 112. (E-F) Unequal separation of chromosomes during telophase. (G-J) Abnormal chromosome arrangement caused by multipolar spindles. (K) Multipolar chromosome segregation. (L) Multipolar cell division. Bar: 10 μm.

Some anomalies, such as mixed-ploidy, were found in the self-pollinated progeny generation of octoploid *B. napus*. The frequency of occurrence of mixed-ploidy individuals in the self-pollinated progeny population of octoploid *B. napus* was between 1% and 3%. Several different types of mixed-ploidy plants were detected by flow cytometry analysis (Figure S11). The mixed-ploidy plants were mostly aneuploids, as confirmed by somatic chromosome counting (Figures 8, S12). There was no obvious integer relationship between the chromosome numbers of different cytotypes (Figures 8, S12). Due to the slight asymmetry and partial overlap of the flow cytometry peaks (Figure S11), the results of flow cytometry could not fully reflect the true chromosome numbers of the mixed-ploidy plants: the results obtained by somatic chromosome counting were far more complicated than the corresponding flow cytometry results (Figures 8, S12). Moreover, there were a variety of mitotic abnormalities in the cells of mixed-ploidy plants, including lagging chromosomes during mitotic metaphase, unequal chromosomal distribution to daughter cells, abnormal chromosome arrangements caused by multipolar spindles, and chromosome loss (Figure 9). Therefore, mitotic instability appears to further exacerbate the genomic instability of the mixed-ploidy plants.

## Discussion

Genome duplication can affect plant phenotypes and physiology in various ways (reviewed by Doyle and Coate 2019), but the effects may vary significantly by taxa and ploidy level. Although polyploidy generally leads to increased organ size, with larger, more vigorous plants (Ramsey and Ramsey 2014), this is not always the case. We observed a decrease in vegetative organ size in our autoallooctoploids relative to the allotetraploid *B. napus*. Similar results were also found in chromosome-doubled *B. napus* (Olsson 1960b) and *B. juncea* (Olsson 1960a), where general plant height and size were reduced relative to the allotetraploid parents. Abel and Becker (2007) had similar findings in synthetic autotetraploid *B. rapa* and *B. oleracea*, and attributed this effect to inbreeding depression caused by increased homozygosity. Hence, crossing between different autooctopoloid lines may be of interest in restoring “hybrid vigour”: (Gowers 1977) also had some success with this approach in tetraploid turnip breeding, where crosses between two inbred autotetraploid lines showed increased biomass in many cases relative to the homozygous parent autotetraploids. In addition, the octoploid *B. napus* plants are relatively short, with strong lodging resistance, large flowers and abundant pollen, and thus could have good ornamental potential.

Our results indicated that additional WGD significantly altered the structure of the male reproductive organs, specifically resulting in an increase in the number of pollen apertures in octoploid *B. napus* relative to tetraploid *B. napus*. This finding supports the hypothesis that a ploidy-sensitive mechanism affects the establishment of pollen apertures (Reeder *et al.* 2016). The germination apertures are where water and air enter and exit the pollen grain, and where the pollen tube extends out (Edlund *et al.* 2004). Pollen grains with three germination apertures are ubiquitous in dicotyledonous plants, including the *Brassica* genus (Reeder *et al.* 2016). Changes in the aperture number may affect the ability of pollen grains to regulate volume changes and to be involved in reproduction (Albert *et al.* 2018). Pollen tubes of octoploid *B. napus* pollen with four apertures could grow faster than those of tetraploid *B. napus* pollen with three apertures, but encountered more difficulties during *in vivo* fertilization, which was in line with the view that an increase in the number of germination apertures could be conducive to competition of pollen on the stigma but not conducive to fertilization (Prieu *et al.* 2016). Thus, four or more apertures may affect the chance of successful fertilization in higher-order dicotyledonous polyploids. We confirm that recurrent polyploidization can drive novel traits in *Brassica* evolution.

Polyploidy is a problem for the normal completion of mitosis and meiosis due to the complex chromosome pairing that can occur when more than two copies of each chromosome are present (Comai 2005). Meiotic stability is a key hurdle that must be overcome following WGD (Pelé *et al.* 2018) and is one of the hallmarks of an adapted polyploid (Baduel *et al.* 2018). Meiotic instability in newly synthesized *B. napus* has been extensively documented, and found to be due to frequent homeologous chromosome pairing between the A and C sub-genomes (Gaeta *et al.* 2007; Xiong *et al.* 2011; Szadkowski *et al.* 2011). The meiotic response to additional WGD of an established allopolyploid has not previously been reported. In this study, it was found that WGD of allopolyploid *B. napus* did not majorly disrupt meiotic stability and in particular pollen viability. Despite many meiotically abnormal behaviors, pollen mother cells of octoploid *B. napus* (AAAACCCC) mostly formed tetrads, the majority of which successfully developed into normal pollen grains, with pollen viability per plant up to 90%. Similar results of high pollen fertility were observed by (Olsson 1960a) and (Olsson 1960b) in genome-doubled *B. juncea* and *B. napus*, supporting our finding. Resynthesized *Brassica* hexaploids (AACCCC (Li *et al.* 2013) and AAAACC (McNaughton 1973)) resulting from crosses between *B. rapa* and *B. napus* also possessed fairly stable meiotic behavior in most pollen mother cells, with pollen fertility of approximately 90% (Li *et al.* 2013; McNaughton 1973). However, in autooctoploids formed from the diploid species, including autooctoploid *B. rapa* (AAAAAAAA, 2*n* = 8*x* = 80), autooctoploid *B. nigra* (BBBBBBBBBB, 2*n* = 8*x* = 64), and autooctoploid *B. oleracea* (CCCCCCCC, 2*n* = 8*x* = 72), the meiotic process was extremely disordered, pollen grains were severely deformed, and pollen viability was reduced to 45.4%, 2.1% and 36.8%, respectively (Liu 2014). More seriously, the majority of pollen grains of autooctoploid *A. thaliana* were dead at anther dehiscence (Wang *et al.* 2010). It appears that higher-order allopolyploids have a stronger ability to regulate meiotic stability and to form normal pollen than higher-order autopolyploids. This suggests that higher-order allopolyploids have greater reproductive capacity and adaptability than higher-order autopolyploids, supporting the view that allopolyploidy results in higher adaptive potential than autopolyploidy (Van de Peer *et al.* 2017). Interactions between sub-genomes may also play an important role in maintaining meiotic stability and pollen viability in higher-order polyploids. In resynthesized *Brassica* allohexaploids (AABBCC), meiotic stability and fertility vary based on parental species origin and genotype (reviewed by Gaebelein and Mason 2018). For instance, some allohexaploid genotypes from the cross *B. carinata* × *B. rapa* are immediately meiotically stable (Gupta *et al.* 2016), while the majority are unstable (Tian *et al.* 2010), while striking differences in fertility and stability have been observed between allohexaploids derived from the cross *B. juncea* × *B. oleracea* (Mwathi *et al.* 2020; Zhou *et al.* 2016), *B. napus* × *B. nigra* (Gaebelein *et al.* 2019a) and (*B. napus* × *B. carinata*) × *B. juncea* (Gaebelein *et al.* 2019b; Mwathi *et al.* 2017). The meiotic stability of allohexaploid AABBCC may be affected by both interactions between the A, B and C sub-genomes, as well as specific genetic loci (Gaebelein *et al.* 2019b; Zhou *et al.* 2016). The regulation of meiotic stability of octoploid *B. napus*, which contains two sub-genomes in two copies each, may fall somewhere between that of a true autopolyploid and a true allopolyploid.

Newly formed polyploids may be particularly common in angiosperms, but most polyploid plants are predicted to go extinct shortly after formation, such that few successfully establish as a new species (Leitch and Leitch 2008; Soltis *et al.* 2014; De Storme and Mason 2014; Wang *et al.* 2010). Although the gametophytes of our octoploid *B. napus* were vigorous, they still encountered certain difficulties during the reproductive process. Fertility-related traits (silique number, seed number, silique length) of the octoploid *B. napus* were significantly lower than those of tetraploid *B. napus*, but the octoploid *B. napus* could successfully set hundreds of self-pollinated seeds with an average of eight seeds per silique, which was enough to propagate the next generation. This phenomenon appeared consistent over several generations. Our resynthesized higher-order octoploids were propagated and maintained for 7 generations of self-pollination, indicating that the post-pollination reproductive barriers were not extremely serious during this self-pollination process. We hence propose based on these results that the octoploid *B. napus* has achieved reproductive success over a short evolutionary period. Similarly, previous studies found relatively good fertility in hexaploid *Brassica* composed of the A and C sub-genomes (AACCCC (Li *et al.* 2013) and AAAACC (McNaughton 1973)) in the first three generations. Meanwhile, some hexaploid *Brassica* AAAACC plants also failed to set seed (McNaughton 1973), similar to observations in autooctoploid *Brassica* (Liu 2014). Some resynthesized *Brassica* allohexaploids (AABBCC) are also highly fertile, but with some sterile plants within each generation (Mwathi *et al.* 2017; Mwathi *et al.* 2020; Gaebelein *et al.* 2019b). Whether the fertility in octoploid *B. napus* in our study is related to sub-genomic interactions or to particular genetic combinations is unclear.

Although several rounds of genome doubling have occurred in the evolutionary history of the *Brassica* genus, the highest ploidy level observed in natural *Brassica* species is only tetraploid, indicating that *Brassica* lineages have the tendency and ability to downsize their duplicated genomes over a long period of time (Hohmann *et al.* 2015). It has been proposed that three strategies of genome evolution (shrinkage, expansion, or equilibrium) are possible for organisms to find an optimal balance between genomic stability and plasticity (Schubert and Vu 2016); of these strategies, genome contraction appears to be the most common (Hufton and Panopoulou 2009; Mun *et al.* 2009; Wendel 2015; Leitch and Leitch 2008; Yang *et al.* 2011). However, genome size variation and changes in the early generations of newly formed polyploids can be complicated even within a single genus. The genome sizes of self-pollinated offspring of the resynthesized octoploid *B. napus* were normally distributed, with the maximum being several times the minimum, and each of genome expansion, contraction and equilibrium being observed. However, there was a general trend toward loss of chromosomes over gain of chromosome copies in the early generations of allohexaploid *Brassica* (AABBCC) (Zhou *et al.* 2016; Gaebelein *et al.* 2019b). The chromosome numbers of offspring of newly formed tetraploid *B. napus* were maintained at or near 38 (Xiong *et al.* 2011). Our results indicate that additional WGD may increase the plasticity and flexibility of the polyploid genomes, thereby effectively buffering against the unbalanced numbers of chromosomes and gene copies, and thus allowing plants that deviated from euploidy to survive. This finding is in line with the view that the success of newly formed angiosperm polyploids is partly attributable to their highly plastic genome structure (Leitch and Leitch 2008). Moreover, the chromosome loss in the self-progeny of octoploid *B. napus* seemed to be more likely to occur in the C sub-genome, which was consistent with the previous finding that the A sub-genomes are more stable than the C sub-genomes (Zhou *et al.* 2016). Besides, the octoploid *B. napus* appears to have the ability to rapidly change genome size, which may promote the acquisition of novel traits and aid in species diversification (Puttick *et al.* 2015), and which enabled us to observe some highly divergent lineages.

Chromosomal instability is common after WGD in both natural and synthetic lines, and extensive aneuploidy is commonly observed in early generation neopolyploids (De Storme and Mason 2014; Soltis *et al.* 2016; Chester *et al.* 2012; Zhou *et al.* 2016; Xiong *et al.* 2011). Possibly due to factors such as tetrasomic sub-genome dosage, homeologous chromosome pairing and chaotic segregation of chromosomes, a large number of aneuploids with an extremely wide range of ploidy variations were involved in the early generations of synthesized octoploid *B. napus* and accrued across multiple generations. This observation is in full agreement with previous studies and further strengthens the view that polyploidy and aneuploidy have played an important role in the evolution of the Brassicaceae (Warwick and Alshehbaz 2006). Additionally, chromosome instability may also result from the inability of cells to handle aberrant behaviors during mitosis (Hufton and Panopoulou 2009; Comai 2005). Although genomic instability such as multiple spindles has been reported in plant meiosis (Risso-Pascotto *et al.* 2005), abnormal mitosis in polyploids has rarely been directly studied. We found clear evidence of genomic instability during mitosis in *Brassica* polyploid plants. We found severe cases of unequal segregation of chromosomes and multipolar spindles in mitosis cells of self-pollinated progeny of octoploid *B. napus*, possibly due to the higher number of chromosomes passing through the threshold for error-free segregation in mitotic cell division (Comai 2005). This phenomenon could also be due to sub-genomic chromosome imbalance; similar situations have been observed in aneuploid and polyploid human cancer cells (Ganem *et al.* 2009). These mitotic abnormalities increased the chance of chromosome mis-segregation and the formation of cytotypes with a series of chromosomes, consistent with the discovery of mixed-ploidy descendants of octoploid *B. napus*. Mixed-ploidy plants may play a bridging role in the formation of plants with different ploidy levels (Varela-Álvarez *et al.* 2018). However, mixed-ploidy plants have seldom been reported in *Brassica*, such that our findings of multipolar spindles in mitosis and mixed-ploidy plants in the self-pollinated offspring of higher-order octoploid *B. napus* provide insights into the diversity and complexity of the *Brassica* genome and the role of ploidy variation in adaptation.

## Conclusions

In this study, we assessed the effects of additional WGD on extant allotetraploid *B. napus*. WGD decreased the size of vegetative organs but slightly increased the size of reproductive organs in allotetraploid *B. napus*. Despite some abnormal chromosomal behaviors, the octoploid *B. napus* exhibited fairly high meiotic stability and produced about 90% viable pollen. The extra WGD significantly changed the appearance of the male reproductive organs of *B. napus*: in particular the number of pollen apertures of octoploid *B. napus* increased from three to four. The octoploid *B. napus* demonstrated generally moderate seed fertility, and could set several hundreds of seeds per plant on average. Higher ploidy is not necessarily a barrier to reproductive success in *Brassica*, or possibly in induced autopolyploids of allopolyploid species in general. The genome size of self-pollinated progeny of the octoploid *B. napus* varied greatly, and was accompanied by extensive genomic instability, such as aneuploidy, mixed-ploidy and mitotic abnormality. The additional WGD may have amplified the tolerance of *B. napus* to genome instability. The octoploid *B. napus* could go through any of genome reduction, equilibrium or expansion in the short-term, thus providing a novel library of karyotypes for the *Brassica* genus. Our results provide novel insights into the early stages of recurrent polyploidization events and the short-term evolutionary fate of newly-synthesized octoploid rapeseed, as well as supporting an extremely high degree of plasticity in *Brassica* genomes. Additional WGD in present allopolyploid species could drive novel traits or features, and in future, related studies in other genera may help to improve our understanding of the evolution of polyploid angiosperms.
